# The use of formative research to inform the design of a seasonal malaria chemoprevention intervention in northern Nigeria

**DOI:** 10.1186/s12936-016-1526-9

**Published:** 2016-09-15

**Authors:** Clare E. Strachan, Musa Kana, Sandrine Martin, John Dada, Naome Wandera, Madeleine Marasciulo, Helen Counihan, Maxwell Kolawole, Tanimu Babale, Prudence Hamade, Sylvia R. Meek, Ebenezer Baba

**Affiliations:** 1 Malaria Consortium, Development House, 56-64 Leonard Street, London, EC2A 4LT UK; 2 London School of Hygiene and Tropical Medicine, Keppel Street, London, WC1E 7HT UK; 3 Department of Community Medicine, Kaduna State University, Kaduna, Nigeria; 4 EPI Unit, Institute of Public Health, University of Porto, Porto, Portugal; 5 Inspire Sustainable Development Agency, Baden Powel House, Buganda Road, Kampala, Uganda; 6 Katsina State Ministry of Health, State Secretariat Complex, IBB Way, Katsina, Nigeria

**Keywords:** Community perceptions, Drug delivery, Formative research, Intervention design, Malaria, Nigeria, Preventive treatment, Qualitative, Seasonal malaria chemoprevention

## Abstract

**Background:**

Experience of seasonal malaria chemoprevention (SMC) is growing in the Sahel sub-region of Africa, though there remains insufficient evidence to recommend a standard deployment strategy. In 2012, a project was initiated in Katsina state, northern Nigeria, to design an appropriate and effective community-based delivery approach for SMC, in consultation with local stakeholders. Formative research (FR) was conducted locally to explore the potential feasibility and acceptability of SMC and to highlight information gaps and practical considerations to inform the intervention design.

**Methods:**

The FR adopted qualitative methods; 36 in-depth interviews and 18 focus group discussions were conducted across 13 target groups active across the health system and within the community. Analysis followed the ‘framework’ approach. The process for incorporating the FR results into the project design was iterative which was initiated by a week-long ‘intervention design’ workshop with relevant stakeholders.

**Results:**

The FR highlighted both supportive and hindering factors to be considered in the intervention design. Malaria control was identified as a community priority, the community health workers were a trusted resource and the local leadership exerted strong influence over household decisions. However, there were perceived challenges with quality of care at both community and health facility levels, referral linkage and supportive supervision were weak, literacy levels lower than anticipated and there was the potential for suspicion of ‘outside’ interventions. There was broad consensus across target groups that community-based SMC drug delivery would better enable a high coverage of beneficiaries and potentially garner wider community support. A mixed approach was recommended, including both community fixed-point and household-to-household SMC delivery. The FR findings were used to inform the overall distribution strategy, mechanisms for integration into the health system, capacity building and training approaches, supportive interventions to strengthen the health system, and the social mobilization strategy.

**Conclusions:**

Formative research played a valuable role in exploring local socio-cultural contexts and health system realities. Both opportunities and challenges for the introduction of SMC delivery were highlighted, which were appropriately considered in the design of the project.

## Background

In many countries in the Sahel sub-region of Africa, malaria transmission is notably seasonal, with most of the disease burden occurring during a brief rainy season lasting 3–4 months [1]. In recent years, seasonal malaria chemoprevention (SMC), previously referred to as intermittent preventive treatment in children (IPTc), has emerged as a priority malaria control intervention due to its high protective efficacy when targeting *Plasmodium falciparum* malaria in children under 5 years old in areas of highly seasonal transmission.

The aim of SMC is to prevent malarial illness by maintaining therapeutic anti-malarial drug concentrations in the blood throughout the period of greatest risk of infection. The intervention involves a complete treatment course of sulfadoxine-pyrimethamine plus amodiaquine (SP + AQ) to children aged between 3 and 59 months at monthly intervals, beginning at the start of the transmission season, to a maximum of 4 monthly courses [1].

SMC was formally recommended by the World Health Organization (WHO) in 2012 [1] for implementation by national malaria programmes across the Sahel sub-region, in response to a growth in evidence on feasibility of delivery, the intervention’s safety, effectiveness and cost effectiveness [3–15]. SMC was also incorporated into the WHO global technical strategy for malaria 2016–2030 [2]. The Nouakchott Initiative was signed in May 2013 by the six country governments of The Gambia, Chad, Mali, Mauritania, Niger and Senegal in order to accelerate and coordinate regional malaria control efforts, with a focus on SMC as a key strategy [16]. The geographic scope was later expanded to include Burkina Faso and Nigeria, with the SMC focus in the latter on the nine northern States [17]. Ten countries across the Sahel sub-region have now incorporated SMC into their strategic plans for malaria control.

There are several potential approaches to delivering SMC, though there remains insufficient evidence to recommend a standard deployment strategy for achieving high coverage given the diverse contextual realities of communities to be served [1, 9]. Approaches best suited to local conditions are recommended, with delivery assuring the continuum of care within the public health system [1, 18]. This facilitates local ownership of the intervention, important for sustainability purposes, as well as to enable care for children with fevers suspected to be malaria and the reporting of any adverse events during SMC administration. An integrated approach also enables the strengthening of the public health system’s reporting of malaria cases and related deaths, and capacity for pharmacovigilance and drug resistance monitoring. In particular, studies have indicated the effectiveness, as well as cost effectiveness, of deploying SMC or IPTc through community health workers (CHWs) in different settings [9, 18–20] though they suggest that coverage and adherence to the monthly treatment regimen can be affected by the specific delivery approach used. Experience of implementing SMC through CHWs has further emphasized the importance of a functional community health system, the consistent involvement of regional and district health authorities, participation of community members in sensitization and mobilization prior to SMC initiation, and the provision of appropriate incentives for both CHWs and participating health facility personnel [9, 10, 15, 18, 20, 21]. There is comparatively less evidence available relating to health-facility based delivery of SMC, though experience has suggested challenges in achieving high coverage of targeted children through a fixed facility-based approach [21].

In 2012, a three-year SMC project was initiated in Katsina state, northern Nigeria, with the aim of exploring the feasibility, acceptability, effectiveness and costs of introducing a scalable community SMC delivery system. With SMC already incorporated into national malaria control policy, this intervention was intended to inform the development of national SMC implementation guidelines and scale up plans within the Nigerian public health system. Katsina state was selected due to its location in the seasonal transmission zone, the availability of community-based health volunteers to enable community delivery of SMC, and the presence of remotely located populations with limited access to formal health care who were considered a useful test case for the proposed delivery system.

In order to be both effective and accepted by communities, it was important that the design of the delivery approach carefully considered both the specific socio-cultural environment and health system realities. Some past experiences of community health interventions in northern Nigeria have been challenged by suspicion when communities perceived programmes to be ‘new’ or ‘external’. Religious and cultural beliefs have been shown to be important in determining the way people understand disease; the high levels of refusal of the oral poliovirus vaccine were reportedly due to poor disease risk perception and incompatible cultural and religious understandings [22]. Katsina state also faces a number of significant challenges to health service delivery, though these are not unique to Katsina and can be seen in states across the country. These include a fragmented health service delivery, inadequate and inefficient financing, weak health infrastructure, inadequate and mal-distribution of the health work force and weak coordination among key players [23]. At the community level, a permanent CHW cadre formally linked to the health system does also not exist in Nigeria, though, as in Katsina, community volunteers are often recruited for specific public health campaigns (such as mosquito net distributions or mass drug administration) and thus have a broad experience in community health care delivery. So as to effectively consider this local context, the SMC project in Katsina was initiated on the back of formative research (FR). This paper describes the FR findings and implications for the design of the SMC delivery approach that was adopted by stakeholders.

## Methods

### Study setting

Katsina, located in far northern Nigeria and bordering Niger to its north, has an estimated population of 6.5 million people (national census projections, 2015). As in other northern Nigerian states, malaria is endemic in Katsina with all year round transmission at levels below national averages, with a seasonal peak (60 % of annual malaria cases) between the months of August and November, coinciding with the raining season (unpublished health system data). Project implementation planned to target four of the 34 Local Government Areas (LGAs) within Katsina state; Baure, Dutsi, Mai Adu’a and Mashi. The LGAs have a collective population of 868,752 (national census projections, 2015) and are found on the northern fringe of the state where malaria prevalence is reportedly higher. In total, there are 1427 health facilities in the state, constituting primary health centres, maternal and child health centres, health clinics and dispensaries [23]. The population is largely engaged with subsistence farming, living predominantly in widely dispersed settlement clusters. Islam is the dominant religion in the area and Hausa and Fulani are the predominantly spoken languages.

### Study objectives

The aim of the FR was to explore the local socio-cultural and health system context and to highlight information gaps and practical considerations to inform the design of an effective SMC delivery approach, inclusive of supportive interventions. Specifically, the objectives were to explore; (1) existing services, including scope and perceived quality, available for managing malaria at the health facility and community level, to which the SMC intervention could be linked, (2) community level health-seeking behaviour patterns, related attitudes, beliefs and other influencing factors, (3) the feasibility of different SMC delivery approaches, including at health facility level, community fixed point, or household-to-household, and (4) any socio-cultural or health system related factors which could either support or hinder the delivery of SMC.

### Study design and participants

The study was qualitative in design and involved a series of in-depth interviews (IDIs) and focus group discussions (FGDs) among 13 pre-identified target groups. All groups were considered active participants in project planning, delivery or evaluation, and/or were direct beneficiaries of the project, and together were expected to represent an optimum range of opinions and perspectives. At the state level, the groups included government policymakers, LGA management and health officials, and United Nations (UN) agencies or non-governmental organizations (NGOs) who were engaged in work of a relevant scope. Health facility representatives included either committee chairpersons of Health Facility Management Committees (HFMCs) or health facility in-charges. At the community level, targeted groups included community leaders, Village Health Committee (VHC) representatives, male household heads, mothers and female caregivers, health-orientated Community Based Organizations (CBOs), as well as ‘Community Caregivers’ (CCGs). As already discussed, a range of community health volunteers exist in Nigeria with varying names, including role model caregivers, community drug distributors and health mobilizers, depending on the scope of donor support and focus of public health interventions being delivered i.e. health promotion, malaria treatment for children, mass drug administration for neglected tropical diseases or immunization campaigns. During early discussions with the Katsina authorities, it was decided that the community members to be involved in supporting SMC delivery would be called ‘Community Caregivers’ so as to distinguish them from other community level health workers. Specific CCG participants in the FR were selected at random. Finally, nomadic groups were not specifically targeted for enquiry; according to Katsina state informants, approximately 1 % of the state population are expected to be nomadic, mostly arable farmers entering Katsina from other states or Niger during the rainy season and moving largely without their families (and thus children who could be targeted for SMC), or pastoral farmers largely moving in the dry season. However, opportunities and challenges for including nomadic populations within the SMC target population were discussed as part of the research.

### Scope of enquiry

The scope of enquiry was developed through a landscape review of relevant published and grey literature and through discussion with experts, including project staff and Katsina state stakeholders. Linked to the FR objectives, three thematic parameters were identified against which specific research questions were developed (Table 1).

**Table 1:** Technical scope of enquiry

Thematic parameter	Questions to be answered from formative research
Health system context	What is the functionality and perceived quality of health care at the health facility level, specifically for the management of malaria?
	What is the scope of health services offered and perceived quality of care at the community level for the management of malaria, including referral?
	How functional are the links between the community and health facility levels and how does this affect quality of care?
	How could access to quality malaria treatment at the community level be improved, including for referred patients?
	What is the existing precedence for financial or other support to CCGs?
	What is the capacity for and existing level and quality of reporting by CCGs? Do they see reporting as important?
Socio-cultural context	What is the level of knowledge, range of attitudes and care-seeking patterns in the community with regards to malaria prevention and treatment? What are these affected by?
	What is the relationship between the CCGs and the community, including levels of support to and trust in CCGs?
	What written and spoken languages are best understood? What are literacy levels like and how could this affect the development of communication materials or the feasibility of caretakers of children under five to keep simple diaries on fever cases and adverse events?
	What information and communication channels could best support promotion and uptake?
	How do community leaders or other figures influence health related behaviours? How could the project best collaborate with them in order to raise acceptability and uptake?
	Are there other ways in which community support could be garnered?
SMC delivery approach	How feasible, acceptable and effective could different drug delivery methods be i.e. health facility, community fixed-point, or a household-to-household? What challenges could be anticipated and how could these be addressed?

### Data collection

Data was collected during March 2013, four months ahead of the first cycle of SMC delivery. IDIs or FGDs were selected as appropriate for each target group; informants were grouped together where it was considered this would likely enhance the quality of opinion provided across respondents, whilst not adversely influencing respondent candour. All interviews were semi-structured in nature and guided by pre-tested topic guides, ensuring a focus was retained on the pre-agreed scope of enquiry (specific questions as relevant for each target group) while enabling new and potentially unexpected perspectives or ideas to be raised. The sample size was set at between 50 and 60 IDIs/FGDs a priori, considered appropriate for enabling deep-case orientated enquiry across target groups whilst remaining manageable within the time and budget available. Sampling within target groups was largely purposive, with specific wards, village and households selected at random for target groups active at these levels (Table 2). At village level, the random walk approach was used given no updated household lists were available.

**Table 2:** Target groups, data collection and sampling

Target group	Interviews conducted	Sampling
Katsina State Government	4 IDIs	All personnel with key roles in malaria control delivery
LGA leadership	4 IDIs, I FGD	At least 1 representative from each LGA
LGA primary health care and malaria focal staff	5 IDIs	At least 1 representative from each LGA
UN agencies, active international and local NGOs	5 IDIs	All representatives with key roles in malaria control delivery
Health facility representatives (committee chairpersons of HFMCs) or health facility in-charges	8 IDIs	2 representatives per LGA, from both primary and secondary levels
Traditional and religious community leaders	6 IDIs	Including both traditional and religious leaders. 2 informants per LGA were originally targeted. Specific wards were selected at random
Village Health Committees (VHCs)	2 IDIs, 5 FGDs	2 villages were selected at random within the ward selected at random (same ward as for community leadership IDIs). All members of the VHC invited to participate
CCGs	2 IDIs, 3 FGDs	All CCGs in each LGA were invited to participate but selected at random if a large number. A target of 1 FGD/LGA. IDI if only 1 participant
Health-orientated CBOs	2 FGDs	All representatives with key roles in supporting community-level health interventions
Household heads (male participants)	3 FGDs	2 villages were selected at random within the ward selected at random (same villages selected as for VHCs). Specific households were selected at random via the random walk method, with the interval selected dependent on village size. Original target of 2 FGDs/LGA
Caregivers of children under five (female participants)	4 FGDs	2 villages were selected at random within the ward selected at random (same villages selected as for VHCs). Specific households were selected at random via the random walk method, with the interval selected dependent on village size. Original target of 2 FGDs/LGA
Total	36 IDIs, 18 FGDs (54)	

The research team included two field teams of researchers with supervisors, one overall field investigator (MK) and two research advisors (EB, CS). The field level team was comprised of equal numbers of men and women, considered important in a patriarchal setting such as northern Nigeria to facilitate both access to households and the disclosure of personal views by women. All field level team members had prior experience in qualitative data collection in a rural Nigerian setting, possessed relevant language skills and were familiar with the specific socio-cultural setting. They all attended a four-day training workshop on the study aims, process and tools, based in Katsina.

All interviews were conducted where possible in quiet places, where the likelihood of interruptions or overhearing was considered minimal. All participants in each FGD were comprised of one sex only so as to reduce inhibitions in the sharing of opinions. Interviews were conducted in English or Hausa depending on the target group. For those conducted in Hausa, the transcripts were written in Hausa and then translated into English, with regular spot checking on clarity and meaning by field supervisors for quality control. All interviews were conducted by two researchers, one conducting the interview and the other writing notes of key points raised for back-up. All interviews were audio-recorded and verbatim transcriptions were developed preferably the same or the following day in order to enhance data validity, and the consideration and capture of interview context, as well as to enable the point of data saturation to be more effectively determined whilst in the field.

### Data analysis

Thematic analysis followed the ‘framework’ approach [24] whereby a pre-existing coding frame was developed based on the scope of enquiry (deductive approach) to which codes were added on review of the data (inductive approach). All data were coded and indexed in Excel (Microsoft).

### Intervention design process

The FR preliminary findings were presented at a one-week long ‘intervention design’ workshop held in Abuja during April 2013. Attendees included key representatives from the national level (political and health leadership and the National Malaria Elimination Programme) and state level (political leadership, malaria focal people, primary health coordinators, health educators and management staff), as well as key NGO and SMC project specific staff. The representation of key health system stakeholders was important for building a sense of local ownership and ensuring consistency with national policy and guidelines. Plenary discussions and group work were used to build consensus on key decisions and the next steps in the design process in four agreed technical areas; (1) design of delivery system (including case management and pharmacovigilance), (2) capacity building, (3) advocacy, communication and social mobilization, and (4) monitoring and evaluation. The process for incorporating the FR results into the project design was iterative in that further questions arose both during and after the workshop, with further clarifications sought from the detailed findings once analysis was complete or through discussion with key stakeholders.

### Ethical considerations

The Katsina State Health Research Committee granted ethical clearance for the study in March 2013 (linked to system by date: letter dated 5 March 2013). Informed oral consent was acquired from all IDI and FGD participants. The consent form included information on the broad aims of the study, confidentiality, respondent rights and uses of the data, and was translated into Hausa. No interviewees refused to participate in the study. Participant identifiers were removed from documentation at the interpretation stage of the data analysis.

## Results

### Study participants

In total, 54 interviews (36 IDIs and 18 FGDs) were conducted across all target groups, as outlined in Table 2, which met the overall target sample size of 50–60 interviews. Sampling details are also provided; any gaps between actual numbers interviewed and targeted numbers is explained by little opportunity to conduct repeat visits to follow up on unavailable interviewees given the tight timeframe of the FR. The point of data saturation was considered by the research team to have been reached.

### FR findings

This section discusses key findings from the FR considered most pertinent to the design and planning of the SMC delivery approach and supportive interventions.

#### Health system context

##### Perceived quality of care

Community level respondents generally described the quality of care at health facility levels as *“good”* and “*satisfactory*”, with positive emphasis given to strong staff commitment and effort in often “*challenging circumstances*”. Criticism was focused on (in order of most commonly raised) insufficient staffing, inadequate staff skills (often linked to the use of casual staff), a limited service range, low capacity to manage severe malaria, inconsistent drug availability (in particular of artemisinin-based combination therapy [ACT]), and the high cost of malaria treatment (treatment is free for pregnant women and children under five only and in the case of stock outs, patients tend to pay for treatment from private pharmacies). The higher health facility levels seemed to evoke more negative comments, seemingly because expectations for technical competence and quality of care were higher. State respondents also raised the issue of black market sale of drugs at health facilities during periods of stock outs, though the extent of this practice was unclear:

*Because the government doesn’t provide the drugs, drugs are being purchased by the healthcare facility staff and sold to patients. If he (facility staff) has a patient, he will not care to refer him because he wants money he and does not like to release the patient.* (State level respondent, IDI)

The scope of health services offered by community health volunteers generally was unclear to most target groups, perhaps unsurprising given the varying scope in roles and the lack of official, routine link with the formal health system. State, LGA and health facility level respondents tended to describe community health volunteer roles as covering mobilization, accompanying parents to health facilities and community management of fever, whereas community members emphasized the sharing of information on health tips and the distribution of drugs, with little expectation for them to conduct any clinical examination or diagnosis. The inconsistent supply of ACT at the community level also appeared to confuse understanding over the role of community health volunteers in the management of uncomplicated malaria. State level respondents also highlighted how inconsistent financial and programmatic support, tied to independent campaigns, was affecting sustainability of community health volunteers in general.

In general, community members indicated a good level of satisfaction with community level care, despite the gaps and inconsistency in service provision. Community health volunteer “*kinship ties*” with the community and close proximity to households reportedly made them a common first point of care. However, health facility and other higher level stakeholders raised a lack of appropriate training among community health volunteers, low access to diagnostic tests, a lack of support for referral and inconsistent availability of ACT. A subtle mistrust of community health volunteers was also apparent which appeared to arise from an overlap of roles with facility level staff, and a lack of clarity in supervision arrangements (see below):

*[The community] feel very happy for the service by us because they are even coming to our houses [to] collect medicine. Even when we go to their houses, they are very friendly and nice to us.* (CCG, FGD, Mai’Adua)

*The quality of care at the community level depends on their capacity. I think it is just satisfactory.* (State level respondent, IDI)

##### Linkage with the health system

There were mixed reports regarding health facility ‘supervision’ provided to community health volunteers, again likely to be dependent on specific arrangements under individual public health campaigns. In some areas, health facility staff support to community health volunteers appeared limited to drug distribution whilst in others, there appeared to be some additional supervisory components. Both health facility and CCG respondents mostly described the relationship as ranging from “*good*” to “*cordial*”. Health workers at both state, health facility and community levels emphasized an inefficient data flow system as well as unclear reporting requirements, with many claims of either a lack of community level reporting duties, unclear reporting lines, or infrequent or irregular reporting by community health volunteers to health facility or state levels:

*[The community health volunteers], they bring out the report, sometimes they do not and we used to tell them to be submitting reports. (*Health facility in-charge, IDI Mai Adu’a)

*They only supervise us as when they give us drugs*—*that is what it is about*. (CCG, FGD, Baure)

Referral linkage between community and health facility levels appeared to be weak, with respondents across target groups mentioning poor documentation (referral notes are not routinely available), a poor feedback mechanism, prohibitive costs borne by patients for transportation and food requirements, and bad roads:

*There is very poor quality in referring. The system lacks proper and clear documentation across the board. [There is a need] to provide referral forms because they normally use exercise books which don’t provide feedback to either side within the referral system.* (LGA leadership, IDI, Mai’Adua)

Some communities appeared to have access to a public ambulance for which patients are required to cover the cost of fuel, whilst others relied on private transport for referral. Community-initiated fundraising efforts for which funds can be channelled towards referrals appeared to be relatively widespread.

On a general level, there was existing precedence and support for the provision of incentives to support community health volunteer activity, though specific stipend amounts have varied by campaign. There was however a difference of opinion across target groups as to whether incentives should be given to CCGs for their role in the SMC drug distribution. Some suggested allowances for training, meals, travel or fuel, as well as general monthly “*facilitation*” could boost motivation, whilst others thought this would raise suspicion for suggesting the need to ‘buy’ people’s involvement indicating the project may not be for the ‘community good’:

*They can be saying why the incentive and they can begin to suspect. They may say ‘most likely now they have not succeeded with polio, now they want to start with malaria again’ and you may even find people sending message that ‘it contains this and that is why it is being given free of charge to the people’. The most important thing is that to educate the people let them understand, this malaria is there and everybody has experienced it. (State level respondent, IDI)*

#### Socio-cultural context

##### Health-seeking behaviour

Communities appeared to have a good general understanding of the signs and symptoms of malaria and the groups most at risk. Care-seeking decisions were seemingly led by knowledge of drug availability, perceived effectiveness of drugs, proximity to household, trust in the provider and associated costs of seeking treatment. The majority of community respondents said they would ideally first seek care from community health volunteers given their close proximity and familiarity, followed by chemists or other private vendors and finally health facilities or hospitals. Self-medication and management of suspected malaria with “*paracetamol*” and “*sponging*” before formal advice or care was sought appeared to be common practice. Opinions of male household heads ultimately appeared to influence household care and treatment decisions, which was expected given the patriarchal society in Katsina state and the dominant role of men as custodian of household financial resources.

*Interviewer: Like in this town, whom do you trust much and listen to?*

*Caregiver 1: My husband.*

*Caregiver2: District head.*

*I: Why is this?*

*C2: Because district heads are together with our husbands.*

*C1: He is the one to grant the permission on everything*. (Caregivers, FGD, Mai Adu’a)

There appeared to be little community level demand for malaria diagnosis, with the focus remaining on access to free drugs whether malaria was confirmed or suspected. Findings indicated a high acceptability of ACT and injections for treatment of severe malaria.

It was suggested that the population of ‘mobile-fixed groups’ within the targeted intervention area was larger than had previously been expected, though specific population estimates varied. It was indicated that malaria prevalence can be proportionately higher among these groups compared with settled communities, yet their health-seeking at formal health facilities tended to be lower:

Y*es, we know the [nomadic] population here is about 1600 people. We have about ten settlements. Generally malaria affects nomadic population because they are living near the small ponds where they give their animals drinks. They access health care through mobile and outreach activities.* (Health facility worker, IDI, Baure)

##### Mobilization and community support

All groups emphasized that community support for the intervention would be encouraged by the involvement of key community stakeholders, in particular community health volunteers (in this case CCGs) as well as traditional and religious leaders, in the mobilization and sensitization pre-delivery as well as the actual delivery of drugs. Religious and traditional leaders such as “*Dagaci*” and “*Imam”* are seen as gatekeepers to the community and “*trusted bearers*” of health and other information. They frequently play a role in supporting community level dissemination of health information by health workers:

*You know, it is our tradition here that our people do not disrespect their leaders, and they are living in peace, that is why it is very difficult for them to disobey them, especially when they give them certain information about something…Honestly, they trust their leaders right from district head, village and ward heads.* (Community leader, IDI, Baure)

*The masses always believe in their traditional and religious institutions, because they are the ones that are always closer to the people and they know their problems and most people do solve their financial and social problems from their advice.* (VHC, FGD, Mashi)

Town criers, village development committees, public functions such as weddings and naming ceremonies, and media such as radio and television were also mentioned across target groups as key communication channels for announcing plans for forthcoming community activities.

*The majority of people listen to radio so it is important especially*—*the FM and state radio, it is good, a lot of jingles, a lot of message should be passed through these media houses and secondly, there is need when you see the community leaders, they should call their own village heads to have meeting so that they can disseminate information given to them, down to the grassroots*. (State level respondent, IDI)

While the data indicated lower levels of community literacy than expected, particularly among women, literacy appeared to be highest for Hausa written in Arabic script and it was suggested that materials available with this writing would likely raise acceptability. Given the low literacy, the ability of caregivers of children under five to keep simple diaries on fever cases and other information, such as adverse events, was deemed questionable, though most community respondents expressed confidence in mothers’ capacity to follow drug administration instructions.

##### Support for the intervention

Informants across target groups thought that the communities would likely be supportive of a SMC programme given tackling malaria reflects a community priority, the drugs would be free, and people have confidence in ACT effectiveness (given the “*few side effects*”). It was suggested that support would be boosted if the drugs were distributed at the most opportune time, specifically when the malaria burden is highest at the onset of the rainy season, and if efforts were taken to effectively engage with community members and leaders. Community respondents also emphasized the importance of “*fulfilling promises”,* given people are conscious of being deceived which can affect long-term support for programmes. However, some informants felt that the negative experience of the polio campaign could override any positive programmatic efforts and enthusiasm for the project:

*Malaria is a serious sickness, distorting the smooth running of our lives, and we are all sick and tired at the same time looking for a solution.* (Household head, FGD, Dutsi)

*The community leaders, policy makers should be involved*—*do you know why? This polio has spoiled everything, so any programme you have, people will suspect it.”*(State level respondent, IDI)

*Some people will think that it is just business as usual due to their previous experience of unfulfilled promises with other organizations.* (VHC, FGD, Mai Adu’a)

The importance of emphasizing SMC as a complement to other malaria control efforts (i.e. environmental management, the use of long-lasting insecticide-treated nets [LLINs] and early care-seeking on identification of symptoms) was also raised, so as to avoid confusion and suspicion, and to maintain a comprehensive approach to malaria prevention. Requests for clear messaging on dosages, timing of doses and possible danger signs were commonly made:

*So, we can encourage the community through telling them the importance of this project. They need to understand. You see, they have to live in a very good environment, no stagnant water, no refuse dumping, using the net. They need to understand all the ways of keeping malaria down*. (LGA leadership, IDI, Mai’Adua)

#### SMC delivery approach

There was broad consensus across target groups that a community level SMC drug delivery system would better enable a high coverage of beneficiaries and garner wider community support, as compared with a health facility distribution system. Concerns were raised across informant groups however about the storage capacity and security of drugs at the community level.

There were divergent views though on the most effective drug distribution method. Some respondents favoured a fixed-point delivery system (administering drugs from a central location which could include a school or health facility) for being an *“easier”* option. Others considered a household-to-household approach (administering drugs in the child’s home) to be preferable given the opportunity enabled to reach and engage with a higher number of targeted beneficiaries and so likely resulting in a higher population coverage and better adherence:

*House to work will work because the one we are doing now is house to house. It is better. We will enlighten women that this medicine is very useful to them*. (CCG, FGD Mai’Adua)

The experience of the polio campaign appeared to work as both a supportive and hindering factor. Some respondents felt that familiarity with the household-to-household approach adopted under this campaign would be likely to raise acceptance of the SMC programme if the same delivery approach was used, whilst other warned of negative connotations given the high refusal rates under the polio campaign:

*For me, it is house to house approach, because it has been tested and trusted during fighting for polio campaign. The secret here is that some view it that, if they go to the hospital, they have to buy this and that, so they may likely not go.* (CBO, FGD, Dutsi)

*They may think it is something else, just like the case of polio… So I will not suggest house to house unless [it is] with very good promotion.* (State level respondent, IDI)

A minority of respondents thought that health facilities should take the lead in the distribution of drugs, in particular where clinics are close to communities, seemingly due to assumptions of the higher technical competence of health facility staff and as a means of further encouraging health-seeking behaviour towards established clinics. Interestingly, most health facility staff supported a community level distribution.

*[Health facility staff] have the information on what the drugs are for; they will be able to dispense it out to the patients adequately. They will be able to monitor any problems that may arise as a result of dispensing the drug.* (State level respondent, IDI)

*When drugs are made available, hand them over to the head of personnel in the hospital, after which people should be made aware of the availability of the drugs in question. People should be told on the days and times they are supposed to be coming for such drugs. It is important they get used to going to the facilities.* (VHC, FGD, Mashi)

A few respondents across community, LGA and state level groups suggested a combination delivery system, with areas adopting either a health facility or community level delivery system, depending on the proximity of health facilities to their target communities:

*I think from the experience two*-*way will help, one by delivering these drugs to the facilities, like in Dutsi LGA where they have quite a number of facilities that are closer and can take care of the communities. Secondly, where communities are far from the facilities, you make outreach, or select an area and a village head and supply them. (*LGA leadership, IDI, Dutsi)

While respondents agreed that community leaders should play a pivotal role in the delivery of the programme, there were differing views on the most effective management and supervision of the distribution process. Higher-level stakeholders (state, LGA, health facility) tended to propose management by health facility personnel so as to maintain quality control and reduce the risk of onward sale of drugs or distribution on political grounds. A number of community level respondents suggested quality control could be managed by assigning a community level committee, constituting community and religious leaders and community health volunteers (in this case CCGs), with specific responsibilities “*to make them dependable*”.

*They have to go to CCGs and what is important is to make the community aware and sensitize them that something is coming and it is for their own good. So, if everybody is aware about the introduction of supply, the CCG will work it 100* *%, free*-*minded inside the community.* (LGA leadership, IDI, Baure)

*You cannot work without the facilities at community level but, if I will advise, you have to use the traditional leader to monitor the facilities, for example, if you have drugs to be distributed for a given community, so that drugs should be channel through the facility because it is drugs and people know that anything that has to do with drugs is from the facilities they will value it most. Then use the community leaders to put an eye on what the facilities are doing…to ensure that what is meant for the community has really gotten to them*. (NGO informant, IDI)

It was emphasized by state and LGA level informants that programme planning and delivery should be integrated as far as possible into the public health system, particularly at the LGA level, so as to access critical local knowledge, to promote ownership and support for the programme, as well as to build local capacity.

*Involving the community will be one of the most important steps. The malaria control programme also has staff at [the] LGA and state level*—*they definitely need to be involved because they know the terrain, they know the people in the communities. They have different sources of advocacy groups. The community and the facilities all have development committees*—*I think it is a nice idea to strengthen those committees*. (State level respondent, IDI)

Table 3 includes a summary of supportive factors and potential challenges which were highlighted through the FR and which required consideration during the project design process.

**Table 3:** Summary of supportive factors and potential challenges for consideration in the design of the SMC intervention

Supportive factors	Potential challenges
Malaria is seen as a community priority	Role of CCGs and scope of services offered unclear across community and health facility levels
Communities have a good understanding of the signs and symptoms of malaria, ways of preventing malaria, and the biological groups most at risk	A range in skills and experience in the management of malaria among CCGs
Wide support for a community level distribution of drugs	Health facility staff perceive CCGs to provide low quality of care
Community level support for and trust in CCGs	Weak referral linkage between community and health facility levels
Close proximity of CCGs to beneficiaries which could enable high intervention coverage and facilitate effective follow-up and monitoring of adverse events	Community referral action potentially hindered by perception of inadequate skills among health facility staff, inconsistent ACT supply and potential cost of transport and malaria treatment
High levels of community acceptability of ACT	Differing opinions on the most effective distribution approach—fixed-point or household-to-household
Supportive supervision system between health facility and CCGs established (though weak in some areas)	A lack of consensus over the suggested management of the intervention and potential roles of the health facility and community leadership
High levels of trust in community traditional and religious leadership, and general consensus that they should play a pivotal role in mobilization for the programme	Low storage capacity at the community level
Community leadership frequently involved in disseminating health information to their communities and so have basic health knowledge and local information dissemination systems are established	Potential security issues relating to the distribution of drugs at the community level
Simple, visual communication materials written in local languages could be well accepted	Varying levels of capacity for effective reporting among CCGs
	Low levels of community literacy (particularly among women) which may inhibit understanding of any written guidance or communication materials as well as record keeping capacity
	Potential suspicions of ‘outside’ interventions, exacerbated by negative associations with the polio campaign

### Implications for design

This section discusses how the FR findings were applied into the design of the SMC delivery approach.

#### Delivery system

It was agreed early on in the planning process to distribute the drugs at the community level, given the broad consensus among respondents that this approach would likely be the most effective for achieving high population coverage and generating community support. The SMC drug regimen was to be four monthly cycles of a three-day course of SMC medicines (SP once, AQ once per day × 3 days) at each annual seasonal round. In Nigeria, both artemether-lumefantrine (AL) and AQ constitute first line treatments for the management of uncomplicated malaria though AQ could not be used during the intervention period because of its use in SMC drug delivery.

Given varying opinions on the specific delivery method, alongside the importance of maintaining community support for locally proposed solutions, a mixed distribution approach was agreed: the specific delivery method decided for each community would depend on the size of the community, cost and distance between households. Broadly, a fixed point approach was proposed for larger, more urban areas and a household-to-household delivery approach for smaller, generally more rural areas. Specific distribution approaches were to be determined during discussions between district health officers and community leaders (specifically community level committees where they existed) during the micro-planning phase, at which point sites for fixed point distributions and the number required for the specific community were also agreed. Local opinion and information very much informed decisions on village level delivery systems.

A number of operational decisions were also guided by the FR findings. In response to security concerns raised in relation to the management of stock at the community level, it was agreed that the last point of stock storage would be the nearest health facility (referral point) and CCGs would simply draw stock from the facility on the distribution day. Crowd control was to be addressed under the guidance of community leadership at fixed point distributions. Given the low levels of literacy, particularly among women, it was agreed that the initial idea of caregivers keeping simple diaries to report fever cases or adverse events among targeted children was not feasible and so CCGs were tasked instead with post-treatment follow-up and appropriate referral. With regards to CCG incentives, it was decided to benchmark the CCG stipend amount at the average level provided by other public health campaigns, at $4.50 USD (750 Naira) per person per day.

For sustainability and acceptability purposes, it was important that the SMC delivery system was (and appeared to be) integrated as far as possible into, and led by, the government health system; this point was clearly highlighted during the FR. Steering committees to oversee and supportively supervise the intervention during the macro- and micro-planning, delivery and evaluation phases were set up at the state and LGA levels. Where possible, the SMC delivery system utilized or aligned with existing government-approved processes or strategies, such as drawing on the general health facility worker refresher training curricula for health systems strengthening (HSS), linking SMC drugs to existing drug reconciliation processes, utilizing existing state, LGA and health facility storage arrangements and aligning the SMC advocacy communication and social mobilization (ACSM) strategy with the broader Katsina Malaria ACSM plan.

Based on suggestions that the population of ‘mobile-fixed groups’ was larger than expected within the intervention area, the micro-planning phase required engagement with their local leaders to sensitize them to the project, clarify usual migration patterns, pre-agree dates and locations for SMC drug delivery and to decide on numbers of targeted children (the population of children under five among stable groups was estimated from polio immunization records, considered the best available data, though nomadic groups have reportedly been challenging to target for the polio vaccine).

#### Strengthening the system and capacity building

The quality of care provided at health facilities was an important consideration given the key role to be played by health facilities in supportive supervision of the programme, the ‘mop-up’ of cases from the household-to-household approach, and the case management of referred suspected malaria or adverse events cases. It was also clear that a comprehensive list of all functional health facilities in the intervention area was not available and further exploration was required to inform the scope of HSS activities to be initiated under the project. A ‘health facility capacity assessment’ was conducted, constituting the mapping of facilities in relation to community catchment areas and referral zones, and an assessment of key capacities required for appropriate malaria case management, commodity logistics, monitoring and evaluation, the identification and reporting of serious adverse drug reactions, as well as a review of human resource availability. Approximately ten health facilities (one per political ward) were selected to be targeted for essential HSS support prior to the intervention, focused on health worker capacity, supply chain and information management. Refresher training for health workers spanned a two-day period and covered appropriate malaria case management, use of rapid diagnostic tests (RDTs), pharmacovigilance, referral, reporting and drug accountability. The supply chain component required a review of existing tools including stock cards and documentation of reverse logistics, storage capacity and, in collaboration with the central medical store in Katsina, a review of available and in-pipeline stock of RDTs and ACT to manage referrals. In terms of information management, the extent to which data requirements could be integrated into the existing reporting system was explored, though given the breadth of timely information needed to effectively monitor the intervention, it was concluded that a parallel reporting system should be developed, involving the use of additional registers, tally sheets and summary forms.

Effective supportive supervision of the distribution was considered critical in order to validate registration numbers, effectively target the SMC drugs and to maintain quality control of the distribution process. To strengthen supportive supervision mechanisms, a detailed checklist was developed which noted CCG core competencies relating to administering SMC drugs, the communication of key messages to caregivers, data recording and follow-up actions. The effective conduct of supportive supervision was also incorporated into the SMC training for health facility workers, which spanned assessing CCG performance, providing feedback and identifying opportunities for improvement. For both reporting and supportive supervision, CCGs were clustered into groups of 10–12 with a focal CGG identified for each cluster; it was hoped that if effective, this set up would continue under other public health campaigns.

Community and health facility linkage was highlighted as weak by the FR. Training in appropriate referral from the community level was critical given the need to refer cases of suspected malaria infection not eligible for SMC, as well as of potential adverse events. Health facility referral points were identified in advance based on proximity to communities and fixed-point distribution sites were located close to health facilities, with the idea that community members could carry out the referral themselves. CCGs were also trained to consider appropriate referral support, such as the provision of transport, depending on levels of urgency.

The findings indicated a good level of satisfaction among community members of community level care led by health volunteers, despite the gaps and inconsistency in service provision, which did not match the perceptions among health facility and other higher level stakeholders of relatively poor community level health care; it was clear benchmarks of ‘quality’ were different at community and health facility levels. The FR also confirmed a wide range of views as to the roles of community health volunteers. It was critical that the roles in relation to the SMC programme be clarified and communicated clearly before the training and as such, standard operating procedures were developed to clarify roles of both CCGs and health facility workers as relating to the pre-, during and post-distribution phases. Roles and responsibilities were to be communicated to community and religious leaders during the social mobilization phase, with the aim of encouraging support for the CCGs given their pivotal role in the programme.

A training curriculum, trainers’ guide and job aids were also developed to raise understanding of, and capacity in relation to, the SMC delivery system for all involved groups. The CCGs’ training covered the eligibility assessment, correct administering of medicines including caregiver home-based administration, referral promotion for ineligible children and the management of adverse events, and use of data collection tools such as the SMC register, patient record card, and referral form. In order to build health facility worker confidence in the capacity of CCGs and support for their role in the distribution of SMC drugs, the training curricula also gave emphasis to the criteria for the community selection of CCGs, in particular their ability to read and write.

A session on inter-personal communication (IPC) skills was also included to support the delivery of accurate, complete and consistent messages to parents and communities as well as to convey a level of professionalism, important for building community and health worker confidence in the programme. The job aid specified key messages to be given to every household to promote compliance with SMC treatment at home as well as to maintain healthy, disease preventative behaviours (i.e. the nightly use of LLINs and early care-seeking). That children taking SMC could still be infected with malaria was also highlighted in the job aid.

#### Communication and social mobilization

Considerable attention was given to overcoming the challenge of low literacy by translating materials and supportive information into Hausa, and Arabic script as recommended, developing simple formats and instructions and using visual guidance where possible. Specific effort was made to develop a set of key messages on SMC using ‘everyday language’ to enable consistent, simple messaging across all communication and social mobilization activities. A unique SMC logo was designed to help low-literacy audiences identify the intervention and differentiate it from other health campaigns such as immunization days; it featured a shield protection from a mosquito which was marked with four tablets, a child at the centre of the logo conveying the target group, and rain indicating when the activity would take place.

The social mobilization strategy gave priority to IPC channels via the community leadership, CCGs, town announcers and ‘word of mouth’ in order to build local ownership, support and momentum for the SMC intervention. The strategy aimed at avoiding aggressive media campaigning which could be perceived as intrusive, or ‘forcing’ people to accept an externally imposed intervention.

The sensitization phase was also integrated into the micro-planning phase and centred around the traditional leadership using a cascade approach. An initial meeting with the state *Emir*, the most senior traditional leader, was followed by a series of meetings with traditional leaders down the hierarchal traditional leadership structure (district heads within target LGAs, and village heads within political wards in the intervention area), to entrust traditional leadership with the dissemination of messages. Leaders received ‘talking points’ in Hausa language to avoid information loss or distortion. Transparency and accountability were given key emphasis, with questions and clarifications encouraged at each level and responses provided by government health system staff so as to emphasize governmental support and integration into the health system. Community leaders were engaged into opening the trainings and micro-planning meetings.

## Discussion

The initial design of the SMC delivery system in Katsina state relied heavily on the FR findings either directly or to highlight gaps which required further information gathering with which to guide the design and the macro- and micro- planning processes. The FR findings were used to inform the overall distribution strategy, mechanisms for integration into the health system, capacity building and training approaches, supportive interventions to strengthen the health system, and the social mobilization strategy. That the scope of enquiry was developed through consultation with key local stakeholders and review of documentation relating to interventions of similar technical and/or geographic scope enabled the capture of information pertinent to the design of an effective SMC programme in this setting. Decisions were made using a data driven approach and involved key stakeholders, most importantly national and state level government personnel, critical for promoting sustainability, ownership and integration into the health system. The broad consultation of different groups to be involved in some way in the intervention enabled due consideration of a range of opinions which could accumulate as either supportive or hindering factors for the SMC programme; awareness of these enabled a design built on opportunities, in particular lines of influence, and which addressed potential challenges upfront. This was particularly important in northern Nigeria given the suspicion which has arose in relation to interventions seen as externally imposed, such as the oral poliovirus vaccine.

A number of potential limitations should be considered. The nature of this enquiry could have given rise to a number of possible biases not uncommon in qualitative research. Specifically, opinions on the quality of care within the health system could have been skewed by respondents not wanting to criticize the work of fellow community members (community health volunteers or health facility workers). The possibility of interviewers influencing responses (moderator bias) should be considered, though was minimized by the careful selection of researchers, the conduct of quality training and emphasis on quality control during data collection. Concept test bias, relating to reaction to new ideas before the processing of consequences or practicalities, may have also been apparent though it was expected in any case that the community would have been broadly supportive of the proposed intervention given controlling malaria appeared to be a community priority. Despite efforts to carefully explain SMC and what the intervention would involve (via standard communication in the topic guide), the concept proved challenging to grasp across respondents, particularly at the community level, with some respondents interpreting SMC as the routine availability of drugs for malaria cases. Despite efforts to encourage confidential and active participation in the interviews, many higher level stakeholders in particular emphasized diplomacy over opinion-sharing and effectively avoided answering a number of questions. The FR also emphasized qualitative methods, considered appropriate for the collection of opinions from a range of stakeholders, though the inclusion of some quantitative elements, such as an objective assessment of quality of care at health facilities, may have been insightful. The short timeframe given to the FR phase, given the forthcoming onset of the rainy season, also meant that the initial design was based on preliminary rather than fully analysed FR findings. However, the iterative nature of the design process also enabled ongoing reflection of the FR findings during and post-analysis.

A growing body of literature advocates for the importance of FR in the design of implementation programmes [25, 26]. This paper adds further evidence to support this message. Some level of formal or informal FR is often done though methods are not usually systematically documented, and the process of applying results into intervention design rarely reported. A retrospective process-orientated end-point evaluation would allow the individual elements of this intervention to be assessed as well as raise overall understanding as to the extent to which the overall design process led to a sustainable and acceptable intervention. The findings from this study are context specific though could be applied in similar settings, particularly as relating to the planning for other public health campaigns in northern Nigeria.

## Conclusions

Formative research played a valuable role in exploring local socio-cultural contexts and health system realities in Katsina state. Both opportunities and challenges for the introduction of a SMC delivery system were highlighted which were appropriately considered in the design of the project.

## Abbreviations

ACSM: advocacy communication and social mobilization
ACT: artemisinin-based combination therapy
AQ: amodiaquine
CBO: community-based organization
CCG: community caregiver
CHW: community health worker
FGD: focus group discussion
FR: formative research
HFMC: health facility management committee
HSS: health systems strengthening
IDI: in-depth interview
IPC: inter-personal communication
IPTc: intermittent preventive treatment for children
LGA: Local Government Area
LLIN: long-lasting insecticide-treated net
NGO: non-governmental organization
RDT: rapid diagnostic test
SMC: seasonal malaria chemoprevention
SOPs: standard operating procedures
SP: sulfadoxine-pyrimethamine
UK: United Kingdom
UN: United Nations
VHC: Village Health Committee
USD: United States Dollars
WHO: Word Health Organization
